# Classification of Muscle Invasive Bladder Cancer to Predict Prognosis of Patients Treated with Immunotherapy

**DOI:** 10.1155/2022/6737241

**Published:** 2022-05-30

**Authors:** Zhifeng Wang, Xiqing Li, Xiaoqing Wang, Jie Liu, Lingdian Wang, Wei Wei, Xiaoyu Duan, Degang Ding

**Affiliations:** ^1^Department of Urology, Henan Provincial People's Hospital, Zhengzhou University People's Hospital, Henan University People's Hospital, Zhengzhou 450003, China; ^2^Department of Oncology, Henan Provincial People's Hospital, Zhengzhou University People's Hospital, Henan University People's Hospital, Zhengzhou 450003, China

## Abstract

**Background:**

Recently, immunotherapies have been approved for advanced muscle invasive bladder cancer (MIBC) treatment, but only a small fraction of MIBC patients could achieve a durable drug response. Our study is aimed at identifying tumor microenvironment (TME) subtypes that have different immunotherapy response rates.

**Methods:**

The mRNA expression profiles of MIBC samples from seven discovery datasets (GSE13507, GSE31684, GSE32548, GSE32894, GSE48075, GSE48276, and GSE69795) were analyzed to identify TME subtypes. The identified TME subtypes were then validated by an independent dataset (TCGA-MIBC). The subtype-related biomarkers were discovered using computational analyses and then utilized to establish a random forest predictive model. The associations of TME subtypes with immunotherapy therapeutic responses were investigated in a group of patients who had been treated with immunotherapy. A prognostic index model was constructed using the subtype-related biomarkers. Two nomograms were built by the subtype-related biomarkers or the clinical parameters.

**Results:**

Two TME subtypes, including ECM-enriched class (EC) and immune-enriched class (IC), were found. EC was associated with greater extracellular matrix (ECM) pathways, and IC was correlated with immune pathways, respectively. Overall survival was significantly greater for tumors classified as IC, whereas the EC subtype had a worse prognosis. A total of nine genes (AKAP12, APOL3, CXCL13, CXCL9, GBP4, LRIG1, PEG3, PODN, and PTPRD) were selected by computational analyses to construct the random forest model. The area under the curve (AUC) values for this model were 0.827 and 0.767 in the testing and external validation datasets, respectively. Therapeutic response rates were greater in IC patients than in EC patients (28 percent vs. 18 percent). Patients with a high prognostic index had a poorer prognosis than those with a low prognostic index. The nomogram constructed from nine genes and stage achieved a C-index of 0.71.

**Conclusion:**

The present investigation defined two distinct TME subtypes and developed models to assess immunotherapeutic treatment outcomes.

## 1. Introduction

Bladder cancer (BC) ranks fourth out of all types of malignant tumors in the male population, causing more than 80,000 new BC cases and about 18,000 BC deaths in America in 2020 [1]. Bladder cancer comes in two varieties: nonmuscular invasive (NMIBC) and muscular invasive (MIBC), and this classification depends on whether tumor cells are confined to the lamina propria or spread into the muscularis propria. About 90% of NMIBC patients can survive more than five years after surgical resection [2]. For MIBC patients, radical cystectomy and chemotherapy are the standard treatments, but only 60% of them can survive for at least five years [3]. Thus, there is urgently needed to develop new therapeutics for MIBC patients.

Immune checkpoint blockade (ICB) has improved cancer therapy, particularly for MIBC [4]. While immunotherapy is approved for the treatment of MIBC, only about 20% of MIBC patients are responsive to the treatment [5]. Multiple biomarkers have been identified to predict ICB response. For example, in many malignancies, tumor mutation burden (TMB) has been proven to be a reliable predictor of immunotherapy response [6]. In a study evaluating pembrolizumab on lung cancer [7], the objective response rate (ORR) was 63% (high TMB) and 0% (low TMB), respectively (*p* value = 0.03). The progression-free survival (PFS) was 14.5 versus 3.7 months, respectively (*p* value = 0.01). However, there are several limitations to the clinical utility of TMB in predicting immunotherapy responses, including the following: (1) TMB testing is costly, time-consuming, and labor-intensive and needs a large sequencing capacity [8]. (2) TMB testing is not standardized for the different assays. (3) The definition of “high TMB” varies across studies. (4) A meta-analysis found that there was no significant difference between high-TMB patients and low-TMB patients in overall survival (OS) [9]. Additionally, the tumor microenvironment (TME), which is composed of immune and stromal cells, may affect the prognosis and clinical effectiveness of antitumor immune treatment [10]. The density of tumor-infiltrating lymphocytes (TILs), especially CD8+ T cells in the TME, is positively associated with the ICB response [11]. As a result, it is critical to get a deep understanding of the inflammatory infiltration features in MIBC.

Other than biomarkers, it is also crucial to provide accurate models to select potential ICB-sensitive patients. Machine learning algorithms that can effectively learn from a given database and provide accurate and reliable predictions in another dataset should be considered. Moreover, dividing patients into distinct molecular subtypes with different drug responses is a feasible method and is gaining popularity [12, 13]. Thus, the identification of molecular subtypes by TME components and the construction of a machine learning prediction model might contribute to the clinical application of ICB.

In the current study, seven MIBC datasets were included and used to identify TME subtypes with distinct immune/stromal cells and prognosis. An independent cohort was adopted to validate the robustness of the constructed TME subtypes. Then, using 9 genes, a random forest model was created to predict TME subtypes. Another dataset containing ICB-treated patients was utilized to confirm the link between TME subtypes and ICB responsiveness. A prognostic index model and two nomograms were constructed to predict the prognosis of MIBC patients.

## 2. Materials and Methods

### 2.1. Data Collection

The MIBC datasets were obtained from TCGA and GEO platforms. The expression matrix of seven datasets: GSE13507 (62 MIBC samples) [14], GSE31684 (78 MIBC samples) [15], GSE32548 (38 MIBC samples) [16], GSE32894 (51 MIBC samples) [17], GSE48075 (73 MIBC samples) [18], GSE48276 (62 MIBC samples) [19], and GSE69795 (34 MIBC samples) [20] were downloaded and selected as the discovery dataset. For the downloaded expression data that contains negative values, we normalized it by the “Min–Max” normalization method. The “Min–Max” normalization translated the highest value into 1, the lowest value into 0, and other values into a numeric value between 0 and 1. The raw count of RNA-seq data and correspondent clinical information for 408 MIBC cases were downloaded from TCGA and selected as the independent validation dataset for evaluation of the constructed TME subtype. The independent dataset for evaluating the connection between the TME subtype and ICB response rate was the IMvigor210 trial, which included 348 BC patients treated with PD-L1 antibody therapy [21]. The mRNA expression and clinical data from these datasets were obtained.

### 2.2. Identification of TME Subtypes

The pathways of extracellular matrix (ECM), CAF, and immune were chosen for the assessment of cell amounts in the TME. The 10 ECM pathways were downloaded from the previous study [22]. The 9 CAF pathways were summarized from previous studies [23–25]. The 16 immune pathways were retrieved from Molecular Signatures Database [26]. Using the gene set variation analysis (GSVA) R program, the mRNA expression matrix of training datasets was converted into a matrix of TME gene sets. Principal component analysis (PCA) was used to detect batch effects among datasets before and after this conversion. Subsequently, unsupervised consensus clustering (CC), a common method used to find potential subtypes among samples, was performed using the “ConsensusClusterPlus” package [27] in the R language. The optimal number of clusters should be the value when the consensus matrix heatmap is clear and sharp, and the relative change value starts to significantly fall.

### 2.3. Differentially Expressed Genes (DEGs)

The *p* value and log2(foldchange) value for each gene in each discovery dataset (GSE13507, GSE31684, GSE32548, GSE32894, GSE48075, GSE48276, and GSE69795) were obtained. To identify robust DEGs, we utilized robust rank aggregation (RRA) [28]. This approach computed significance scores for all genes by combining the *p* values and log2(foldchange) values from seven discovery datasets. The robust DEGs were chosen based on the threshold of *p* value < 0.05 and ∣log2(foldchange) | >0.5. The pathway enrichment analysis of the robust DEGs was conducted by files of “c5.bp.v6.2.symbols.gmt,” “c2.cp.reactome.v6.2.symbols.gmt,” and “c2.cp.kegg.v6.2.symbols.gmt” with a significance threshold of *p* value < 0.05.

### 2.4. Construction of a Classifier for TME Subtype Prediction

To select genes for model construction, univariate Cox analysis and random forest are adopted. Firstly, the univariate Cox analysis was utilized to discover the genes associated with prognosis. Then, the random forest (RF) model, a machine learning method to select optimal biomarker combinations, was applied to identify subtype-related genes that had higher importance values. The retained genes were thought to be subtype-related genes. The following steps were taken to build and evaluate the classifier based on the 9 genes: (1) 60% of the MIBC samples from the seven discovery datasets were used for model training, while the remaining 40% of samples were used for model validation. And this separation was conducted randomly. (2) In the training dataset, the model was trained by 5-fold cross-validation; (3) in the testing dataset (TCGA-MIBC), the prediction accuracy of the model was calculated; (4) the constructed random forest classifier was used to test the association of TME subtype with ICB response in the IMvigor210 study.

### 2.5. Develop and Validate a Prognostic Index Model

The GEO datasets (GSE13507, GSE31684, GSE32548, GSE32894, GSE48075, GSE48276, and GSE69795) and TCGA-MIBC transcriptional data and patient information were pooled and then randomly split into training (60 percent) and testing datasets (40 percent). On the basis of the mRNA expression levels of nine subtype-related genes, a predictive prognostic index model was created using Cox regression analysis. In the training dataset, the model formula was as follows: prognostic index = (*β*1 × mRNA1 expression) + ⋯+(*βn* × mRNA*n* expression). By dividing the samples from both the training and testing datasets into high-index and low-index groups based on their median prognostic index values, the survival curves of groups were plotted using the R program survminer. The timeROC package was used to assess the prognostic index model's predictive ability.

### 2.6. Construction of Predictive Nomograms

The nomogram is commonly used to estimate the outcome of people with cancer. We developed predictive nomogram models for measuring overall survival (OS) in MIBC patients using the R package rms. The first nomogram was constructed using a combination of GEO datasets and TCGA-MIBC datasets. The second nomogram was constructed from TCGA-MIBC dataset. This is mainly because only samples from the TCGA-MIBC dataset have clinical parameters such as pathological stages. Nomograms were evaluated using the C-index, a measure of predictive power.

## 3. Results

### 3.1. Removing the Batch Effects among Discovery Datasets

**Figure 1:**
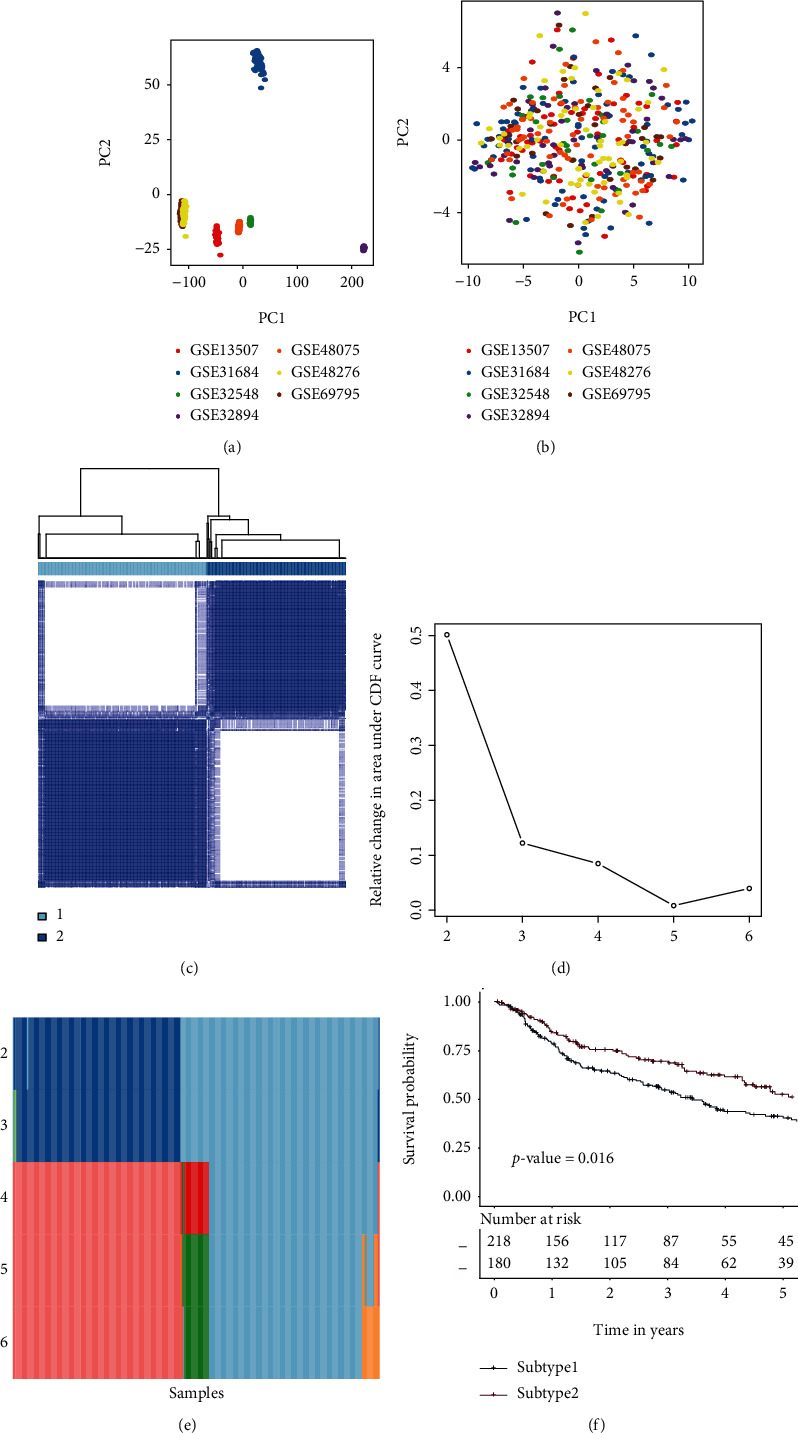
Consensus clustering for MIBC discovery datasets (GSE13507, GSE31684, GSE32548, GSE32894, GSE48075, GSE48276, and GSE69795). (a) PCA of the mRNA expression matrix of 7 discovery datasets. (b) PCA of the TME gene set matrix of 7 discovery datasets. (c) Consensus matrix heatmap of two subtypes. (d) Relative change area values for optimal subtype numbers: 2 to 6. The optimal subtype number in this plot should be the one at which the value starts to drop. (e) The sample distributions from different subtype numbers. The samples in each subtype were illustrated by distinct colors within every row. (f) Subtype-specific survival curves for five-year OS in individuals with MIBC. The log-rank test was used to determine the *p* value among the TME subtypes. Abbreviations: OS: overall survival; MIBC: muscle invasive bladder cancer; PCA: principal component analysis; TME: tumor microenvironment.

A total of 398 samples from seven MIBC discovery datasets with available clinical information were selected and downloaded in this study. Using the GSVA R program, the mRNA expression matrix of training datasets was converted into a matrix of TME gene sets. Before the conversion, there was an obvious batch effect since samples from distinct datasets were isolated from samples from other datasets (Figure 1(a)). According to the PCA plot, the batch effect was successfully eliminated after the conversion (Figure 1(b)).

### 3.2. Identification of the TME Subtypes

To obtain the accurate TME subtypes among 398 MIBC samples from the discovery dataset, consensus clustering was conducted on the matrix of TME gene sets. The tracking plot and delta area determined the clustering number parameter, which ranged from 2 to 6. When *k* = 2, the consensus matrix heatmap showed clean and crisp borders, indicating that the samples were clustered stably and robustly (Figure 1(c)). In the delta area plot, *k* = 2 was selected because its value started to significantly fall (Figure 1(d)). The tracking plot showed that most samples were consistently divided into two subtypes (Figure 1(e)). Thus, *k* = 2 was eventually chosen as the optimal number of clusters after comprehensive consideration. A significant prognostic difference was observed between subtype 1 and subtype 2 (*p* value = 0.029, Figure 1(f)). The consensus clustering was conducted in 408 MIBC samples from TCGA-MIBC by the same parameters. The results from TCGA-MIBC also indicated that 2 was the optimal number of clusters (Supplementary Figure 1A-1C). A significant prognostic difference was observed between subtype 1 and subtype 2 (*p* value = 0.027, Supplementary Figure 1D). Overall, there are two distinct TME subtypes among MIBC samples.

### 3.3. The Relationship between Subtypes and Clinical Characteristics

**Figure 2:**
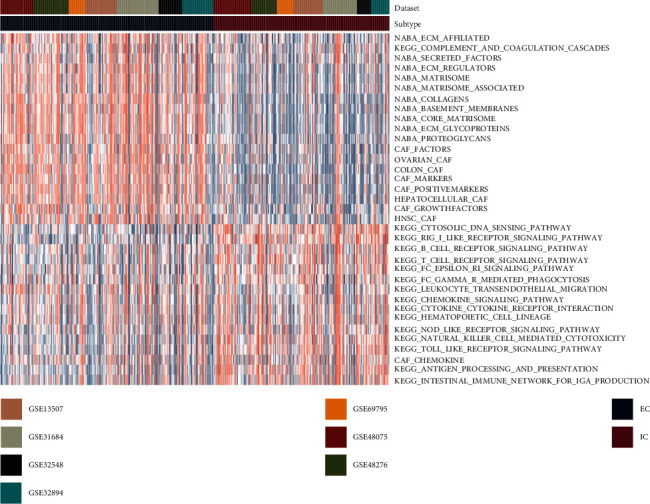
The scores of TME pathways in 2 subtypes from the discovery datasets (GSE13507, GSE31684, GSE32548, GSE32894, GSE48075, GSE48276, and GSE69795). Abbreviation: TME: tumor microenvironment.

ECM and CAF pathways were found to be more prevalent in subtype 1 compared to subtype 2, whereas immune pathways were more prevalent in subtype 2 (Figure 2). Similarly, TCGA-MIBC data indicated that subtype 1 had larger quantities of ECM and CAF pathways, while subtype 2 had greater concentrations of immune pathways (Supplementary Figure 2). Thus, subtype 1 was named ECM-enriched class (EC), and subtype 2 was named immune-enriched class (IC). These results demonstrate that these two TME subtypes are robust and reliable. The relationship between subtypes and clinical characteristics was calculated. And EC was found to be more common in older and advanced-stage patients (Table 1).

### 3.4. Robust DEGs between Two TME Subtypes

**Figure 3:**
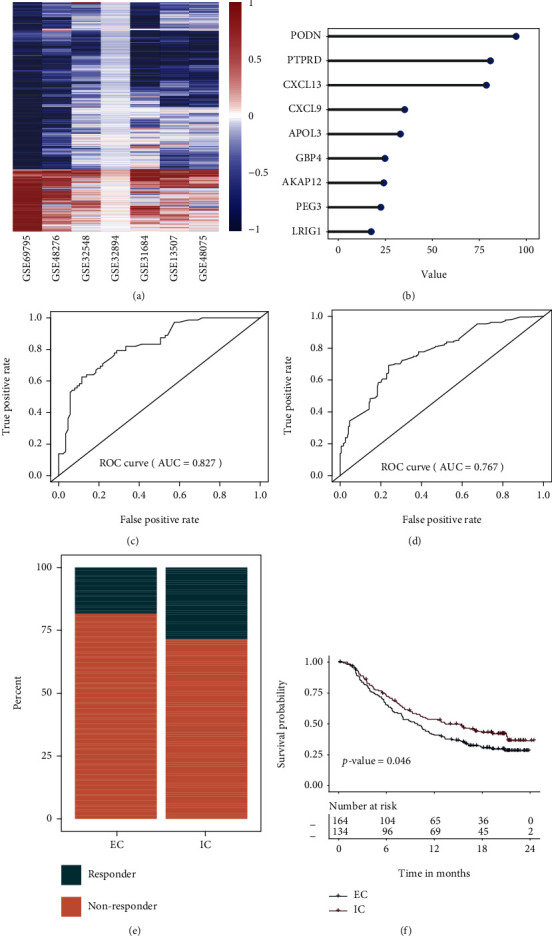
Identification of biomarkers and construction of prediction model. A heatmap depicts the log2(fold change) values of robust DEGs. A row represents a single gene, while a column represents a single dataset. Genes that are upregulated are highlighted in red, whereas those that are downregulated are highlighted in blue. The creation and assessment of a random forest classifier for the prediction of TME subtypes. (a) The 9 genes with the highest importance value were selected for classifier construction in the training dataset. (b) Validation of classifier in the testing dataset. (c) Validation of classifier in the independent validation dataset (TCGA-MIBC). (d) Correlation between TME subtype and therapeutic success rate IMvigor210. (e) Correlation between TME subtype and survival outcome in IMvigor210. DEGs: differentially expressed genes; RRA: robust rank aggregation.

To acquire the robust DEGs in our investigation, two steps were used. To begin, log2(foldchange) and *p* values for each gene in each dataset were calculated. Second, the RRA approach was used to identify a total of 1265 robust DEGs, with 355 increased and 910 deceased genes in subtype 2. The heatmap was used to visualize the combined log2(foldchange) values of selected robust DEGs (Figure 3(a)).

Numerous immune-associated and cell cycle-related biological processes (BPs) were linked to IC, while cell and tissue development processes were found in subtype 1 (Supplementary Table 1). KEGG and REACTCOME were also used to find pathways that were enriched in each TME subtype. Extracellular matrix organization-related pathways were significantly enriched for subtype 1, and immune-associated and cell cycle-related pathways were enriched for IC (Supplementary Table 2, 3).

### 3.5. Machine Learning Model for Predicting TME Subtypes

Survival analysis yielded 120 significant prognosis-related genes, 105 for the positive negative genes (coefficients > 0), and 15 for the positive prognosis genes (coefficients < 0). To create a clinically useful classifier, top informative subtype-associated genes must be chosen. Random forest algorithm was adopted to select the most importance genes. Therefore, the top 9 genes (AKAP12, APOL3, CXCL13, CXCL9, GBP4, LRIG1, PEG3, PODN, and PTPRD) with the highest importance values were selected for classification purposes (Figure 3(b)). The samples from discovery dataset were divided into training dataset (60%) and testing dataset (40%). Then, a 9-gene model was trained by random forest in the training dataset. Additionally, the 9-gene classifier was applied to the testing dataset in order to validate subtype prediction ability (Figure 3(c)), and we observed the AUC of 0.827 in testing dataset. Besides, prediction results from independent dataset (TCGA-MIBC) suggested that the 9-gene classifier can achieve the AUC 0f 0.767 (Figure 3(d)). The process for constructing and validating the constructed prediction model was plotted in Supplementary Figure 3.

### 3.6. Distinct Sensitivity of the TME Subtype to Immunotherapy

Nine-gene classifier and the expression profiles of a published dataset (IMvigor210 cohort) containing 348 cancer patients treated with an immune checkpoint inhibitor (PDL1 antibody) were used. The response rate to immune checkpoint inhibitor therapy was higher in IC than in EC patients (28% vs. 18%) (Figure 3(e)). A significant difference was observed between two TME subtypes, indicating that patients within IC had a better prognosis for immune checkpoint inhibitor therapy (Figure 3(f)).

### 3.7. Construction of Prognosis Model by 9 Genes

**Figure 4:**
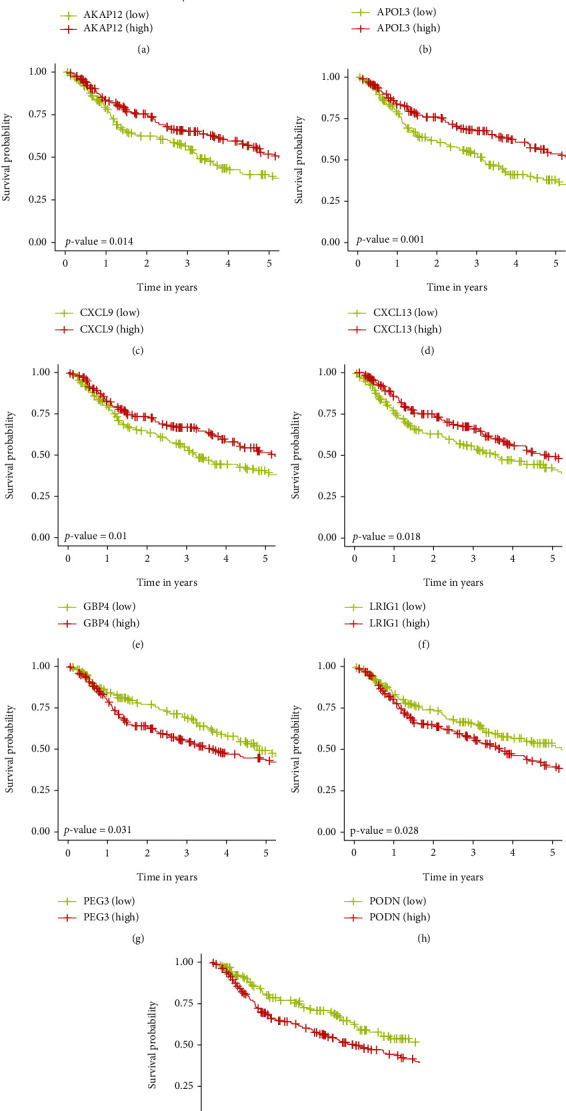
Five-year Kaplan–Meier (K-M) curves for overall survival of MIBC patients in the discovery datasets. The *p* values were calculated by the log-rank test.

The patients from the discovery datasets (GSE13507, GSE31684, GSE32548, GSE32894, GSE48075, GSE48276, and GSE69795) were classified into high and low groups based on the median value of gene expression. Kaplan–Meier plots showed that AKAP12, APOL3, CXCL9, CXCL13, GBP4, and LRIG1 were protective genes (Figure 4). Besides, PEG3, PODN, and PTPRD were found to be risky genes. Similarly, based on the survival data from TCGA-MIBC dataset, APOL3 and CXCL13 were found to be protective genes (Supplementary Figure 4).

**Figure 5:**
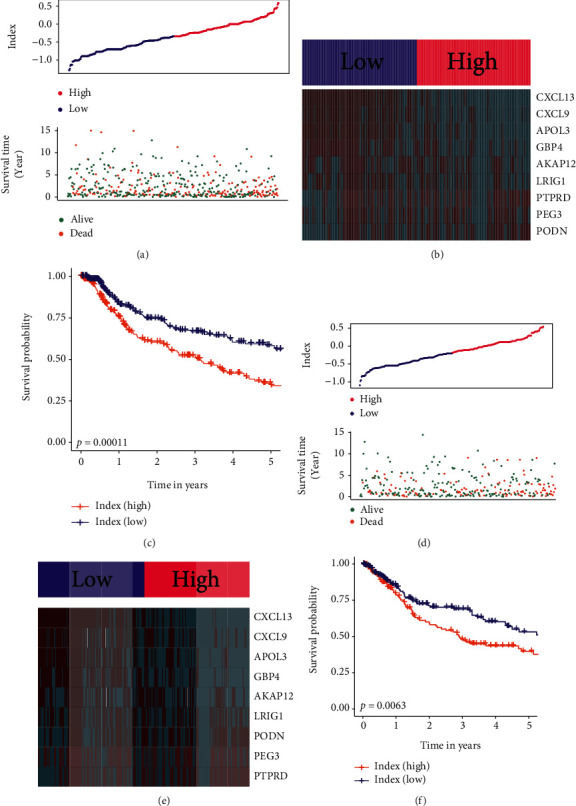
Analysis of prognosis signatures in the training and testing datasets. (a) Distribution of prognostic index and surviving condition in the low and high prognostic index groups from the training dataset. (b) Expression values of genes between two groups in the training dataset. Red color represents high expression value, and green color represents low expression value. (c) Survival curves of the high- and low-index groups in the training dataset. (d) Distribution of prognostic index and surviving condition in the low and high prognostic index groups from the testing dataset. (e) Expression values of genes between two groups in the testing dataset. Red color represents high expression value, and green color represents low expression value. (f) Survival curves of the high- and low-index groups in the testing dataset.

We trained and validated the prognostic model using the seven GEO cohorts and TCGA-MIBC cohort. Firstly, the samples from these eight cohorts were combined into one dataset. The pooled dataset was then separated into training (60 percent) and testing datasets (40 percent) using a randomization technique. The relative coefficients for 9 subtype-related genes were calculated by the multivariate Cox regression model. The prediction model requires the relative coefficient and mRNA expression levels for nine subtype-related genes, as follows: prognostic index = (−0.24)∗AKAP12 + (−0.22)∗APOL3 + (−0.41)∗CXCL13 + (−0.04)∗CXCL9 + (−0.10)∗GBP4 + (−0.12)∗LRIG1 + (−0.15)∗PEG3 + (0.30)∗PODN + (0.28)∗PTPRD. Then, for every observation in the training data, we computed and ordered the index. Thus, participants in the training data set were grouped into two categories: those at low index (*n* = 196) and those at high index (*n* = 195), with the median prognostic index serving as the cut-off value.  Figure 5(a) shows the survival overview and the gene expression profiles. A heat map was created to depict the gene expression patterns of individuals in two index groups (Figure 5(b)). The Kaplan–Meier curves were used to evaluate the survival of high- and low-index groups, and those in the low-index group had a much greater overall survival rate than patients in the high-index group (Figure 5(c), *p* value < 0.001).

### 3.8. Validation of the Prognosis Model

To confirm our findings in the training set, we evaluated the 9-gene signature's predictive ability in the testing dataset. A prognostic index was produced for each MIBC patient in the testing dataset using the same manner as in the training dataset.  Figure 5(d) displays the distribution of prognostic index and a summary of survival in the testing cohort. Additionally, patients in the testing dataset were classified into high-index (*n* = 196) and low-index (*n* = 196) groups based on the median cut-off value. A heatmap was created to depict the gene expression patterns of individuals in two index groups (Figure 5(e)). The survival curve suggested that the low-index group had a better OS compared to the high-index group (Figure 5(f), *p* value < 0.001).

### 3.9. Development of Nomograms

**Figure 6:**
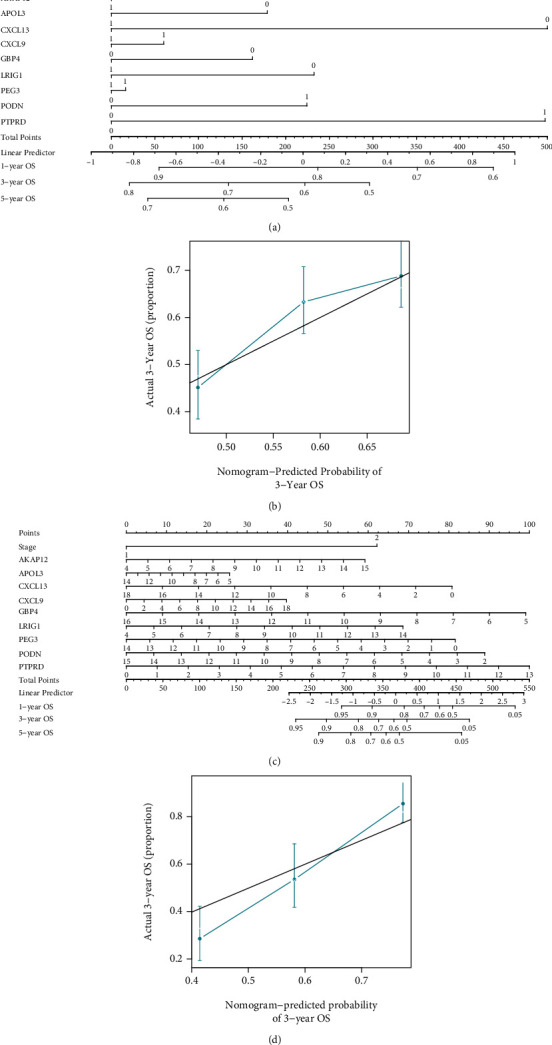
The nomograms. (a) The nomogram constructed by genes in the combination dataset of GEO datasets and TCGA-MIBC cohorts. (b) Calibration lines for 3-year survival prediction in the combination dataset of GEO datasets and TCGA-MIBC cohorts. (c) The nomogram constructed by genes and clinical parameters in TCGA-MIBC cohort. Stages I-II and stages III-IV were represented by “1” and “2,” respectively. (d) Calibration lines for 3-year survival prediction in TCGA-MIBC cohort. Abbreviation: OS: overall survival.

Typically, nomograms are used to quantify an individual's risk. The first nomogram was created to estimate the prognosis of one-, three-, and five-year in patients with MIBC by using the nine genes (Figure 6(a)). The C-index of this nomogram was 0.61, and the calibration curve suggested that actual and expected survival were quite strongly correlated in three-year prognosis prediction (Figure 6(b)). The second nomogram was designed to assess the prognosis of one-, three- and five-year by 9 gene signatures and clinical parameters (Figure 6(c)). And the nomogram suggested that PTPRD plays a more important role than stage in the model. The C-index was 0.71, and the calibration curve of three-year also indicated good prognostic prediction efficacy (Figure 6(d)).

## 4. Discussion

Antibodies that inhibit the interaction of PD1/PDL1 have been authorized for the treatment of a variety of cancers, including metastatic bladder cancer. Due to the fact that the PD1/PDL1 pathway suppresses immune cell responses, inhibiting the PD-1/PD-L1 pathway enables immune cells to attack tumors [29]. However, only a small number (20%) of MIBC patients are responsive to immunotherapy [5], which limits the clinical use and makes it important to select the potential responders. Since multiple studies have discovered that TME has a deep association with ICB response [30, 31], providing a prediction model based on TME to identify ICB responsive patients could have clinical and academic significance. To confirm the robustness of TME subtypes and their connection with ICB response, independent datasets were examined. Our results suggest that there are two TME subtypes with different immunotherapy response rates, and we provided an accurate model for the TME subtype prediction.

The TME that surrounds tumor cells is composed of immune cells, stromal cells, and ECM molecules. TME has significant effects on tumorigenesis and development [32], therapeutic resistance, and clinical outcome. Unlike other studies that only include the immune cells from TME [33, 34], the current study also used the other TME components such as CAF and ECM for subtype construction. In this study, two TME subtypes were found among these seven discovery datasets. EC had higher levels of ECM-related and CAF-related gene sets, and IC had higher levels of immune-related gene sets, respectively. The results suggest that CAF and ECM have a negative association with immune cells, especially T cells. CAFs could directly express PDL1 to induce the exhaustion of T cells [35] and indirectly inhibit T cell function through ECM remodeling that could act as a barrier to block the access of immune cells to cancer cells [36]. Thus, ECM and CAF inhibition might contribute to activating the immune cell and increasing the ICB response.

In this study, we systematically explored the biomarkers related to TME subtypes. A total of 9 genes (AKAP12, APOL3, CXCL13, CXCL9, GBP4, LRIG1, PEG3, PODN, and PTPRD) were selected by DEG analysis, univariate Cox analysis, and importance value calculation in the machine learning model. Results suggest that AKAP12, APOL3, CXCL13, CXCL9, GBP4, and LRIG1 may act as tumor suppressors. On the other hand, PEG3, PODN, and PTPRD may play oncogenic roles. Studies have discovered that AKAP12 acts as an oncogenesis suppressor, for example, AKAP12 deficiency is linked to enhanced metastatic potential in human tumors such as bladder cancer [37]. CXCL9 and CXCL13 are two chemokines that recruit various immune cells. CXCL9 could contribute to the inhibition of angiogenesis and tumor progression by recruitment of T lymphocytes [38, 39]. CXCL13 has dualistic impacts. For example, it can either promote tumor development via PI3K/AKT signaling or improve antitumor immunity by enhancing immune cell invasion [40]. Upregulation of LRIG1 suppresses cell growth and induces cell apoptosis of bladder cancer by inhibiting MAPK and AKT signaling [41].

There are some strengths. Firstly, more than seven MIBC datasets from GEO were used as discovery datasets for the identification of TME subtypes. In the independent dataset (TCGA-MIBC), we discovered the TME subtypes with the same expression pattern and clinical prognosis. This ensures the robustness of the constructed TME subtypes. Secondly, a 9-gene machine learning model was provided in our study. The prediction performance of the model was tested on an independent dataset (TCGA-MIBC). Besides, the association of TME subtypes with ICB response was directly validated by an independent cohort of ICB-treated bladder cancer patients. However, the limitations of this study require mention. Firstly, the identified TME subtypes should be verified by the original study. In the future, we will collect bladder cancer samples with available mRNA expression and clinical data. Then, we will predict the TME subtype for each sample and compare the prognosis difference between two TME subtypes. Secondly, the expression pattern of 9 genes needs to be validated experimentally. Besides, the mechanisms of impact from CAF or ECM molecules on immune cells such as CD8 T cells should be explored further.

## 5. Conclusion

We identified two TME subtypes among MIBC patients, one of them was associated with high levels of immune cells, and the other had more CAF and ECM molecules. A 9-gene random forest model that could predict the TME subtypes was constructed and validated, and this model could serve as a reference for clinical use of ICB.

## Figures and Tables

**Table 1:** Clinical characteristics of TME subtypes from TCGA-MIBC dataset. Abbreviations: TME: tumor microenvironment; MIBC: muscle invasive bladder cancer.

Characteristics	Subtype 1 (EC)	Subtype 2 (IC)	*p* value
	*n* = 176	*n* = 200	
*Gender*			0.881
Female	48 (27.3%)	57 (28.5%)	
Male	128 (72.7%)	143 (71.5%)	
*Age (years)*			0.006
20-50	19 (10.8%)	25 (12.5%)	
50-70	67 (38.1%)	105 (52.5%)	
70-90	90 (51.1%)	70 (35.0%)	
*Stage*			<0.001
Stages I-II	40 (22.7%)	87 (43.5%)	
Stages III-IV	136 (77.3%)	113 (56.5%)	

## Data Availability

The data used to support the findings of this study are available from the corresponding authors upon request.

## Supplementary Materials

Supplementary Materials Supplementary Figure 1: evaluation of constructed TME subtypes in the independent dataset (TCGA-MIBC). (a) Consensus matrix heatmap of two subtypes. (b) Relative change area values for optimal subtype numbers: 2 to 6. The optimal subtype number in this plot should be the one at which the value starts to drop. (c) The sample distributions from different subtype numbers. The samples in each subtype were illustrated by distinct colors within every row. (d) Subtype-specific survival curves for five-year OS in individuals with MIBC. The log-rank test was used to determine the *p* value among the TME subtypes. Abbreviations: TCGA: The Cancer Genome Atlas; OS: overall survival; MIBC: muscle invasive bladder cancer. Supplementary Figure 2: the scores of TME gene sets in 2 TME subtypes from the independent validation dataset (TCGA-MIBC). Abbreviations: TME: tumor microenvironment; TCGA: The Cancer Genome Atlas; OS: overall survival; MIBC: muscle invasive bladder cancer. Supplementary Figure 3: the process for constructing and validating the constructed prediction model. Supplementary Figure 4: five-year Kaplan–Meier (K-M) curves for overall survival of MIBC patients in TCGA-MIBC dataset. The *p* values were calculated by the log-rank test. Supplementary Table 1: the enriched biological process (BP) by gene set enrichment analysis. NES: normalized enrichment score. Supplementary Table 2: the enriched Kyoto Encyclopedia of Genes and Genomes (KEGG) items from gene set enrichment analysis. NES: normalized enrichment score. Supplementary Table 3: the enriched Reactome gene sets by gene set enrichment analysis (GSEA). NES: normalized enrichment score.
