# Supplementary material for: Classification of Muscle Invasive Bladder Cancer to Predict Prognosis of Patients Treated with Immunotherapy
Source: J Immunol Res. 2022 May 30;2022:6737241. doi: 10.1155/2022/6737241 (PMC9170513; doi:10.1155/2022/6737241)
Supplement: Supplementary Materials — Supplementary Figure 1: evaluation of constructed TME subtypes in the independent dataset (TCGA-MIBC). (a) Consensus matrix heatmap of two subtypes. (b) Relative change area values for optimal subtype numbers: 2 to 6. The optimal subtype number in this plot should be the one at which the value starts to drop. (c) The sample distributions from different subtype numbers. The samples in each subtype were illustrated by distinct colors within every row. (d) Subtype-specific survival curves for five-year OS in individuals with MIBC. The log-rank test was used to determine the p value among the TME subtypes. Abbreviations: TCGA: The Cancer Genome Atlas; OS: overall survival; MIBC: muscle invasive bladder cancer. Supplementary Figure 2: the scores of TME gene sets in 2 TME subtypes from the independent validation dataset (TCGA-MIBC). Abbreviations: TME: tumor microenvironment; TCGA: The Cancer Genome Atlas; OS: overall survival; MIBC: muscle invasive bladder cancer. Supplementary Figure 3: the process for constructing and validating the constructed prediction model. Supplementary Figure 4: five-year Kaplan–Meier (K-M) curves for overall survival of MIBC patients in TCGA-MIBC dataset. The p values were calculated by the log-rank test. Supplementary Table 1: the enriched biological process (BP) by gene set enrichment analysis. NES: normalized enrichment score. Supplementary Table 2: the enriched Kyoto Encyclopedia of Genes and Genomes (KEGG) items from gene set enrichment analysis. NES: normalized enrichment score. Supplementary Table 3: the enriched Reactome gene sets by gene set enrichment analysis (GSEA). NES: normalized enrichment score. [file 6737241.f1.zip › Supfigure 2 (1).pdf]

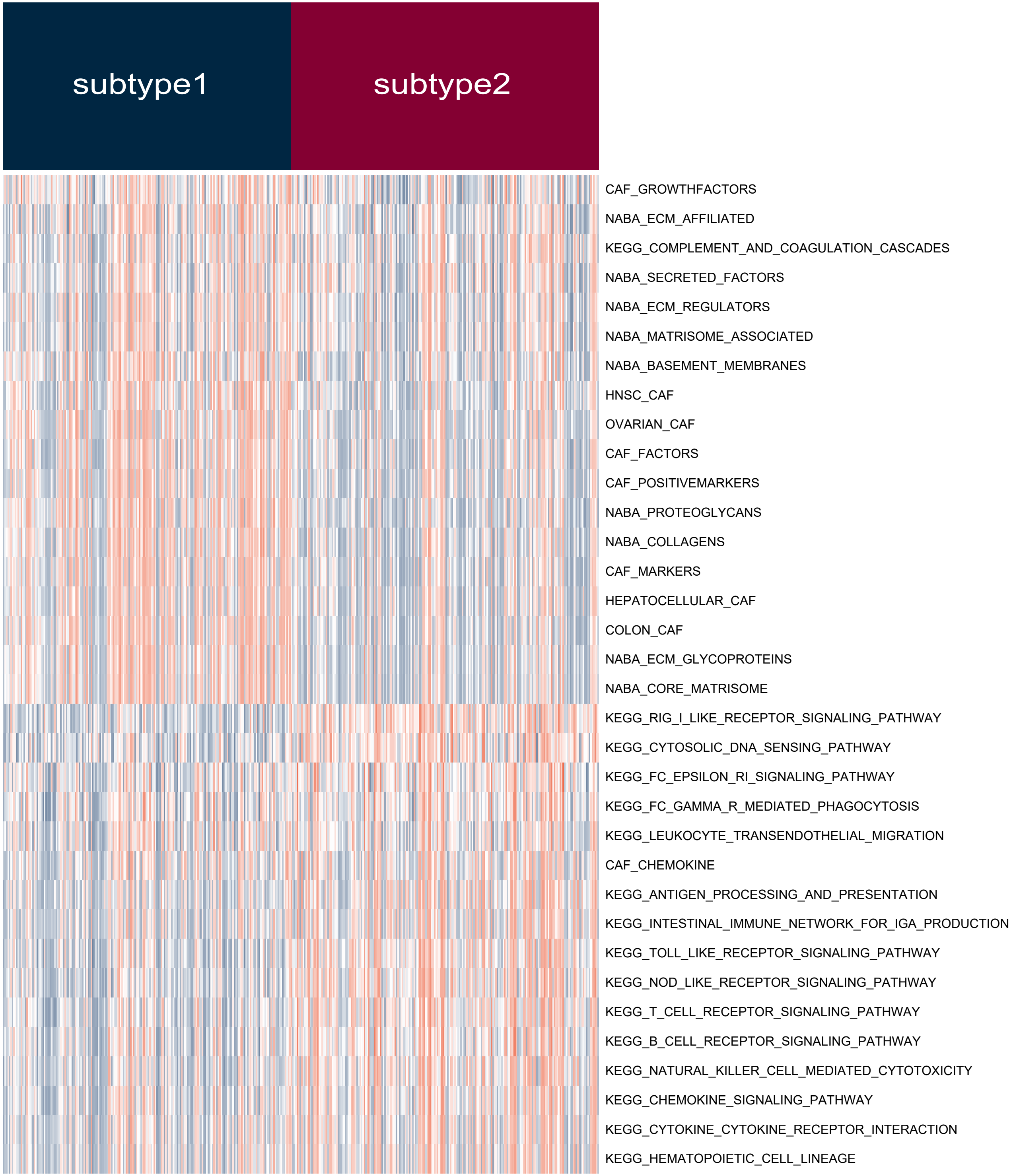
subtype1
subtype2
CAF_GROWTHFACTORS
NABA_ECM_AFFILIATED
KEGG_COMPLEMENT_AND_COAGULATION_CASCADES
NABA_SECRETED_FACTORS
NABA_ECM_REGULATORS
NABA_MATRISOME_ASSOCIATED
NABA_BASEMENT_MEMBRANES
HNSC_CAF
OVARIAN_CAF
CAF_FACTORS
CAF_POSITIVEMARKERS
NABA_PROTEOGLYCANS
NABA_COLLAGENS
CAF_MARKERS
HEPATOCELLULAR_CAF
COLON_CAF
NABA_ECM_GLYCOPROTEINS
NABA_CORE_MATRISOME
KEGG_RIG_I_LIKE_RECEPTOR_SIGNALING_PATHWAY
KEGG_CYTOSOLIC_DNA_SENSING_PATHWAY
KEGG_FC_EPSILON_RI_SIGNALING_PATHWAY
KEGG_FC_GAMMA_R_MEDIATED_PHAGOCYTOSIS
KEGG_LEUKOCYTE_TRANSENDOTHELIAL_MIGRATION
CAF_CHEMOKINE
KEGG_ANTIGEN_PROCESSING_AND_PRESENTATION
KEGG_INTESTINAL_IMMUNE_NETWORK_FOR_IGA_PRODUCTION
KEGG_TOLL_LIKE_RECEPTOR_SIGNALING_PATHWAY
KEGG_NOD_LIKE_RECEPTOR_SIGNALING_PATHWAY
KEGG_T_CELL_RECEPTOR_SIGNALING_PATHWAY
KEGG_B_CELL_RECEPTOR_SIGNALING_PATHWAY
KEGG_NATURAL_KILLER_CELL_MEDIATED_CYTOTOXICITY
KEGG_CHEMOKINE_SIGNALING_PATHWAY
KEGG_CYTOKINE_CYTOKINE_RECEPTOR_INTERACTION
KEGG_HEMATOPOIETIC_CELL_LINEAGE
