# Supplementary material for: Classification of Muscle Invasive Bladder Cancer to Predict Prognosis of Patients Treated with Immunotherapy
Source: J Immunol Res. 2022 May 30;2022:6737241. doi: 10.1155/2022/6737241 (PMC9170513; doi:10.1155/2022/6737241)
Supplement: Supplementary Materials — Supplementary Figure 1: evaluation of constructed TME subtypes in the independent dataset (TCGA-MIBC). (a) Consensus matrix heatmap of two subtypes. (b) Relative change area values for optimal subtype numbers: 2 to 6. The optimal subtype number in this plot should be the one at which the value starts to drop. (c) The sample distributions from different subtype numbers. The samples in each subtype were illustrated by distinct colors within every row. (d) Subtype-specific survival curves for five-year OS in individuals with MIBC. The log-rank test was used to determine the p value among the TME subtypes. Abbreviations: TCGA: The Cancer Genome Atlas; OS: overall survival; MIBC: muscle invasive bladder cancer. Supplementary Figure 2: the scores of TME gene sets in 2 TME subtypes from the independent validation dataset (TCGA-MIBC). Abbreviations: TME: tumor microenvironment; TCGA: The Cancer Genome Atlas; OS: overall survival; MIBC: muscle invasive bladder cancer. Supplementary Figure 3: the process for constructing and validating the constructed prediction model. Supplementary Figure 4: five-year Kaplan–Meier (K-M) curves for overall survival of MIBC patients in TCGA-MIBC dataset. The p values were calculated by the log-rank test. Supplementary Table 1: the enriched biological process (BP) by gene set enrichment analysis. NES: normalized enrichment score. Supplementary Table 2: the enriched Kyoto Encyclopedia of Genes and Genomes (KEGG) items from gene set enrichment analysis. NES: normalized enrichment score. Supplementary Table 3: the enriched Reactome gene sets by gene set enrichment analysis (GSEA). NES: normalized enrichment score. [file 6737241.f1.zip › SupTable 1 (1).docx]

| Pathway | p-value | NES | Size | Type |
| --- | --- | --- | --- | --- |
| GO REGULATION OF MULTICELLULAR ORGANISMAL DEVELOPMENT | 0.002 | -2.44 | 179 | subtype1 |
| GO CELLULAR RESPONSE TO ORGANIC SUBSTANCE | 0.006 | -1.67 | 175 | subtype1 |
| GO TISSUE DEVELOPMENT | 0.002 | -2.53 | 172 | subtype1 |
| GO POSITIVE REGULATION OF RESPONSE TO STIMULUS | 0.018 | -1.61 | 168 | subtype1 |
| GO SYSTEM PROCESS | 0.002 | -1.85 | 161 | subtype1 |
| GO MOVEMENT OF CELL OR SUBCELLULAR COMPONENT | 0.049 | -1.46 | 161 | subtype1 |
| GO CELL DEVELOPMENT | 0.002 | -2.05 | 160 | subtype1 |
| GO REGULATION OF CELL PROLIFERATION | 0.002 | -1.8 | 159 | subtype1 |
| GO POSITIVE REGULATION OF MULTICELLULAR ORGANISMAL PROCESS | 0.002 | -1.95 | 157 | subtype1 |
| GO REGULATION OF CELL DIFFERENTIATION | 0.002 | -2.42 | 155 | subtype1 |
| GO DEFENSE RESPONSE | 0.036 | 1.76 | 116 | subtype2 |
| GO IMMUNE RESPONSE | 0.022 | 2.9 | 85 | subtype2 |
| GO CELL CYCLE | 0.018 | 2.89 | 65 | subtype2 |
| GO CELL CYCLE PROCESS | 0.017 | 2.78 | 58 | subtype2 |
| GO INNATE IMMUNE RESPONSE | 0.02 | 2.47 | 54 | subtype2 |
| GO DEFENSE RESPONSE TO OTHER ORGANISM | 0.016 | 2.39 | 49 | subtype2 |
| GO CYTOKINE MEDIATED SIGNALING PATHWAY | 0.014 | 2.13 | 45 | subtype2 |
| GO MITOTIC CELL CYCLE | 0.014 | 3.38 | 43 | subtype2 |
| GO IMMUNE EFFECTOR PROCESS | 0.014 | 2.27 | 42 | subtype2 |
| GO CHROMOSOME ORGANIZATION | 0.011 | 2.54 | 35 | subtype2 |

**Supplementary Table 1.** The enriched biological process (BP) by gene set enrichment analysis. normalized enrichment score (NES).
